# Mr. Goddard on Fractures of the Cranium

**Published:** 1805-06-01

**Authors:** Francis Goddard

**Affiliations:** Wootton Bassett


### *To the Editors of the Medical and Physical Journal.*

GENTLEMEN,

I AM induced to trouble you with a line on the subject of fractures of the cranium, from an idea that the trephine is oftener used than is really necessary; and if, by any means, we can lessen the pain attending those accidents, without increasing the danger, I think it behoves every surgeon to contribute his observations.

In the course of my practice I have had thirteen cases of fracture in different parts of the cranium, nearly all with more or less depression. In eight of these I did not apply the trephine, and these patients all recovered without any very serious symptoms occurring, and I have known several of them, for years since, enjoying good health.

I will now state two cases, recently occurring. The first, Oct. 18, a farmer, aged 25, rather delicate in make, discharging a gun, it burst, and the breech struck the upper part of the forehead, and depressed a part of the frontal bone, nearly the size of a tea-spoon bowl; he was stunned and beat down, but after a time got home. Next morning, when I was called, I found symptoms of depression, dilated pupil, great pain in the head, sickness and vomiting, slow pulse, and great loss of strength. The wound was about an inch long, and the pericranium was detached from the bone, so that the depression was plainly to be seen. As the case did not appear urgent, I determined to try the effect of bleeding, copious purgings, with a low regimen and a dark room, dressing the wound lightly, and was much pleased to find every appearance go on favourably. The man was able to go out in about three weeks, the sore quite healed, and has remained well ever since.

In the second case, which happened the 22d of November, to a boy, aged 14: As he was lowering a piece of timber into a boat by an engine, the windlas slipped from his hands, and struck him a blow on the parietal bone, about an inch above the ear; a portion of scalp was detached, and the fracture and depression were more extensive than in the first, and so also were the symptoms; however, the success of former cases induced me to try the same plan, of bleeding and a low regimen, and uniting the scalp by the first intention, which could not be done in the former case, as the parts were bruised and burnt by the powder; and am happy in saying every thing went on to my wish; in three weeks the boy boy went home to his friends, and has continued well ever since.

I am well aware the cases above recited are very imperfectly drawn up, and if my practice is generally followed, it will answer no good end to insert them in your Journal. But if they are likely to make the younger practitioners in surgery wait a short time, in many cases, before they use the trephine, that is the utmost of my expectation in sending them to you, and you are at liberty to publish them if you please. I am, &c.

*Wootton Bassett, Jan.* 8, 1805. FRANCIS GODDARD.

